# Characterization of poly-β-hydroxybutyrate and its biosynthesis in *Priestia endophytica* UCM B-5715

**DOI:** 10.55730/1300-0152.2804

**Published:** 2026-04-03

**Authors:** Alina KHARCHUK, Maksym KHARCHUK, Maksym KHARKHOTA, Sergiy ROGALSKY, Oksana TARASYUK, Oleksandr KISTEN, Anastasiia HUBINA, Illya KAPATS, Liliia AVDIEIEVA

**Affiliations:** 1 Department of Antibiotics, Danylo Zabolotny Institute of Microbiology and Virology, National Academy of Sciences of Ukraine, Kyiv, Ukraine; 2 Laboratory of Biological Polymer Compounds, Danylo Zabolotny Institute of Microbiology and Virology, National Academy of Sciences of Ukraine, Kyiv, Ukraine; 3 Polymer Modification Laboratory, Valerii Kukhar Institute of Bioorganic Chemistry and Petrochemistry, National Academy of Sciences of Ukraine, Kyiv, Ukraine; 4 Department of Physiology of Industrial Microorganisms, Danylo Zabolotny Institute of Microbiology and Virology, National Academy of Sciences of Ukraine, Kyiv, Ukraine; 5 Department of Polymers, Faculty of Chemical Technology, University of Chemistry and Technology (UCT) Prague, Prague, Czech Republic; 6 Electron Microscopy Laboratory, Mykola Kholodny Institute of Botany, National Academy of Sciences of Ukraine, Kyiv, Ukraine

**Keywords:** *Priestia endophytica*, polyhydroxyalkanoates, poly-β-hydroxybutyrate, biopolymers, bioplastics

## Abstract

**Background/aim:**

Polyhydroxyalkanoates (PHAs) are biodegradable biopolymers with promising applications in biotechnology and medicine; however, the diversity of their biosynthesis within the genus *Priestia* remains poorly characterized. Although *Priestia* species have been recognized as potential PHA producers, the detailed genomic architecture and physicochemical properties of the polymers synthesized by these bacteria, particularly in the type strain, remain poorly understood. This study aimed to characterize the poly-β-hydroxybutyrate (PHB) produced by *Priestia endophytica* UCM B-5715 and to investigate the PHB biosynthetic genes across related strains.

**Materials and methods:**

PHB granules were visualized and measured using fluorescence microscopy, transmission electron microscopy, and scanning electron microscopy. Polymer structure and molecular weight were examined using Fourier transform infrared spectroscopy, ^1^H nuclear magnetic resonance spectroscopy, and viscometry, whereas thermal behavior was evaluated using differential scanning calorimetry (DSC) and thermogravimetric analysis (TGA). Comparative genomic analysis of 11 *P. endophytica* strains was performed to reconstruct the PHB biosynthetic pathways and to analyze the evolutionary relationships among PhaC synthases.

**Results:**

Microscopy confirmed the intracellular accumulation of PHB granules ranging in size from 65.35 × 47.77 nm to 1544.39 × 1126.53 nm. DSC analysis revealed a glass transition temperature of 2.3 °C, a crystallization temperature of 74.6 °C, and two melting peaks at 155 °C and 164.5 °C, indicating the presence of distinct crystalline domains. TGA demonstrated high thermal stability, with thermal decomposition initiating at 271 °C. Genomic analysis revealed a complete and conserved *pha* gene cluster across all examined strains, with the PhaC proteins being classified as Class IV PHA synthases.

**Conclusion:**

This study provides the first comprehensive physicochemical and genomic characterization of PHB synthesized by the type strain *Priestia endophytica* UCM B-5715, highlighting the potential of this species as a robust microbial source of biotechnologically relevant biopolymers.

## 1. Introduction

Polyhydroxyalkanoates (PHAs) are intracellular polyesters synthesized by diverse microorganisms as carbon and energy reserves. Their intrinsic biodegradability, biocompatibility, and mechanical strength have established them as promising candidates for biomedical applications, including implantable scaffolds and regenerative materials. PHAs exhibit low immunogenicity and degrade into physiologically tolerated intermediates, such as 3-hydroxybutyrate, further supporting their suitability for applications involving direct contact with mammalian tissues (Verlinden et al., 2007; Philip et al., 2007). Additionally, their mechanical properties are comparable to those of conventional petroleum-based plastics (Liang et al., 2024).

The biosynthesis of PHAs exhibits substantial variability among bacteria, with differences in *pha* gene cluster organization, substrate specificity, and regulation. Because the organization of these clusters, particularly that of the PHA synthase gene *phaC*, determines polymer composition and accumulation efficiency, the characterization of unexplored taxa remains essential for expanding the currently known diversity of biopolymer metabolism (Stubbe and Tian, 2003). The identification of new or insufficiently characterized PHA producers may also provide access to strains with unique metabolic capabilities and potentially improved or novel polymer properties (Tsuge et al., 2015).

Current biomedical research continues to integrate PHAs into tissue-engineering platforms. Both pristine poly-β-hydroxybutyrate (PHB) and composite PHA-based materials have demonstrated enhanced osteoconductivity, biocompatibility, and structural stability (Gredes et al., 2015; Codreanu et al., 2020). Reinforcement with components such as hydroxyapatite, polylactic acid, cellulose, or tricalcium phosphate further enhances the mechanical and biological performance of these materials, thereby broadening the applicability of PHAs in orthopedics and dentistry (Karahaliloglu et al., 2015; Kanabenja et al., 2022; Kohan et al., 2022).

Within this context, the genus *Priestia* has emerged as a promising yet still underexplored group of PHA-producing bacteria. *Priestia* species are gram-positive, spore-forming microorganisms commonly inhabiting soils and plant-associated niches, and they display notable ecological versatility and metabolic robustness (Kharkhota et al., 2024). These traits, together with recent evidence of efficient PHA synthesis, distinguish *Priestia* from other gram-positive producers and highlight its industrial potential. *Priestia endophytica*, in particular, has been reported to synthesize both PHB homopolymers and PHB-co-PHV copolymers (Madhusoodanan et al., 2020; Kharkhota et al., 2024). Previous studies demonstrated that the type strain, *P. endophytica* UCM B-5715^T^, accumulates PHB granules and that its pigments, chlorxanthomycin and resistomycin, may be associated with these granules (Kharkhota et al., 2024). However, despite the growing interest in this genus, the genetic determinants underlying PHB biosynthesis and the physicochemical properties of PHB synthesized by the type strain remain insufficiently characterized. Because type strains serve as taxonomic and genomic benchmarks, their detailed analysis is crucial for understanding metabolic capabilities and accurately assessing the biotechnological potential of related isolates.

The present study aimed to characterize the physicochemical properties of PHB produced by *P. endophytica* UCM B-5715^T^ and to investigate the genetic basis and biosynthetic features underlying its production, thereby providing new insights into PHB biosynthesis within this emerging microbial genus.

## 2. Materials and methods

### 2.1. PHB-producing strain and cultivation conditions

The type strain *Priestia endophytica* UCM B-5715^T^ (= DSM 13796), obtained from the Ukrainian Collection of Microorganisms, D.K. Zabolotny Institute of Microbiology and Virology, National Academy of Sciences of Ukraine, was used in this study. The strain was cultured in flasks containing PHA production medium composed of the following components (g/L): peptone, 3.25; NaCl, 2.5; yeast extract, 0.75; and glucose, 20, at pH 7.4 for 24 h at 37 °C.

### 2.2. Isolation and purification of PHB granules

Biomass was collected by centrifugation at 704 × *g* for 5 min and subsequently treated with a 15% (v/v) sodium hypochlorite solution at a biomass-to-solution ratio of 1:100 (m/v) under continuous stirring for 3 h. The lysate was centrifuged at 3835 × *g* for 15 min, and the resulting pellet was washed sequentially five times with distilled water, followed by two washes with 96% ethanol (1:2, m/v). The purified granules were air-dried at 37°C for 48 h.

### 2.3. Preparation of PHB films

A PHB solution (0.07 g/mL) prepared in chloroform was cast onto glass plates. Residual solvent was removed under a vacuum of 10 mbar at 50 °C, yielding films approximately 300 μm in thickness.

### 2.4. Light and fluorescence microscopy

Intracellular PHB granules were visualized using 0.5% aqueous-alcoholic Nile blue staining, as previously described by Kharkhota et al. (2024). Fluorescence microscopy was performed using a green filter, with Nile blue fluorescence detected at 460 nm, as described by Ostle and Holt (1982).

### 2.5. Transmission electron microscopy (TEM)

Ultrathin sections of bacterial cells and isolated granules were prepared using an LKB 8800 ultramicrotome (LKB Instruments, Bromma, Sweden) equipped with a 45° diamond knife (Diatome Ltd., Biel, Switzerland) and subsequently stained with 5% uranyl acetate and 0.4% Reynolds’ lead citrate, as previously described (Bozzola, 2007; Ellis, 2007; Kharkhota et al., 2024). The sections were examined using a JEM-1400 transmission electron microscope (JEOL Ltd., Tokyo, Japan) operated at 80 kV. Granule dimensions were measured from the acquired micrographs using SimpleMeasure software.

### 2.6. Scanning electron microscopy (SEM)

Isolated PHB granules were mounted on carbon adhesive tape, sputter-coated with gold, and examined using a JSM 6060 LA scanning electron microscope (JEOL Ltd., Tokyo, Japan). Granule dimensions were analyzed using ImageJ software (National Institutes of Health, Bethesda, MD, USA).

### 2.7. Gas chromatography–mass spectrometry (GC-MS)

PHB composition was analyzed using an Agilent 6890N/5973 inert gas chromatograph–mass spectrometer (Agilent Technologies, Santa Clara, CA, USA) equipped with an HP-5MS capillary column (30 m × 0.25 mm × 0.25 μm), as previously described by Kharkhota et al. (2024).

### 2.8. FTIR and ^1^H NMR spectroscopy

Fourier transform infrared (FTIR) spectra of PHB samples were recorded using a Vertex 70 spectrometer (Bruker Optics GmbH, Ettlingen, Germany) equipped with a deuterated triglycine sulfate detector and an attenuated total reflectance diamond crystal over the spectral range of 400–4000 cm^−^^1^. ^1^H nuclear magnetic resonance (NMR) spectra of PHB granules dissolved in CDCl_3_ were acquired using a Varian Gemini 2000 spectrometer operating at 400 MHz (Varian Inc., Palo Alto, CA, USA).

### 2.9. Molecular weight determination

The viscosity-average molecular weight (M_v_) of PHB was determined by dilute-solution viscometry using a glass capillary Ubbelohde viscometer, as described by Akita et al. (1976). Polymer solutions were prepared in chloroform at concentrations ranging from 0.10 to 0.24 g/100 mL. Viscometric studies were carried out at 30 °C.

The relative (η_rel_), specific (η_spec_), and reduced (η_red_) viscosities were calculated using the following equations:


$$\eta_{rel}=\frac{t}{t_{0}},\;\eta_{spec}=\frac{t-t_{0}}{t_{0}},\eta_{red}=\frac{\eta_{spec}}{C},$$

where *t*_0_ and *t* represent the flow times of the solvent and polymer solution, respectively, and the reported values correspond to the averages of five measurements obtained for each concentration.

The viscosity-average molecular weight (M_v_) of PHB was calculated using the Mark–Houwink equation:


$$[\eta ]=\text{K}\cdot 10^{-4}\;{\text{M}}^{\alpha },$$

where [η] represents the intrinsic viscosity of the polymer in chloroform, K = 1.18 and α = 0.78, according to Akita et al. (1976).

To determine the intrinsic viscosity [η], plots of reduced viscosity *(η_spec_*/C) and inherent viscosity (*lnη*/C) versus polymer concentration were extrapolated to zero concentration. The accuracy of the measurements was 3%.


$$[\eta ]=\underset{C\to 0}{lim}\frac{\eta_{spec}}{C}=\underset{C\to 0}{lim}\frac{ln\eta_{rel}}{C}$$

### 2.10. Differential scanning calorimetry and thermogravimetric analysis

Differential scanning calorimetry (DSC) analysis was performed using a Q2000 differential scanning calorimeter (TA Instruments, New Castle, DE, USA) over a temperature range of −50 to 250 °C at a heating rate of 20 °C/min during the second heating cycle. Thermogravimetric analysis (TGA) was performed using a Q50 thermogravimetric analyzer (TA Instruments, New Castle, DE, USA) over a temperature range of 20 to 500 °C at a heating rate of 20 °C/min under an air atmosphere.

### 2.11. Genome characterization and reconstruction of the PHB biosynthetic pathway

A total of 11 *P. endophytica* strains, including the type strain UCM B-5715^T^ (= DSM 13796), were included in the comparative genomic analysis. Sequences and annotations were obtained from NCBI. The genome of the type strain was reannotated against the UniProtKB/Swiss-Prot database and analyzed using *merlin* to identify genes involved in PHB biosynthesis and degradation (Capela et al., 2022). Gene–protein–reaction associations were generated using KEGG Orthology, and the metabolic pathways were visualized using KEGG Mapper. Whole-genome alignments were performed using progressiveMauve. The PHA biosynthetic gene clusters were compared with those of *P. megaterium* ATCC 11561 (AF109909) and *B. cereus* YB-4 (AB525763) using pyGenomeViz. The evolutionary relationships among PhaC proteins were inferred using the maximum likelihood method based on the Jones–Taylor–Thornton (JTT) substitution model applied to 28 MUSCLE-aligned sequences (Jones et al., 1992). Initial phylogenetic trees were generated using the Neighbor-Joining and BioNJ methods. A timetree was generated using the RelTime method implemented in MEGA X software (Kumar et al., 2018).

### 2.12. Statistical analysis

Granule dimensions were analyzed using Statistica 12 software (StatSoft Inc., Tulsa, OK, USA) by descriptive statistical analysis, including calculation of the mean, minimum and maximum values, variance, and quartiles. Sample sizes were n = 91 for transmission electron microscopy (TEM) and n = 100 for scanning electron microscopy (SEM) measurements.

## 3. Results

### 3.1. Morphological features of intracellular PHB granules

**Figure 1:**
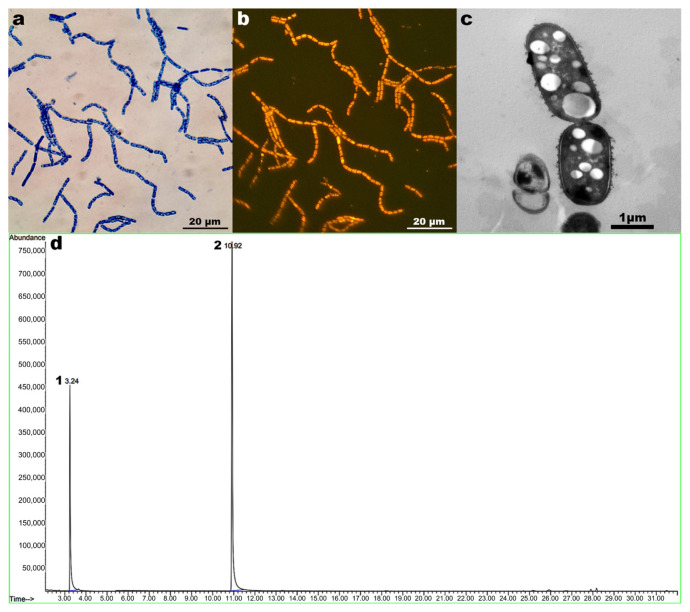
Light (a) and fluorescence (b) micrographs of *P. endophytica* UCM B-5715^T^ cells showing Nile blue-stained PHB granules; TEM of ultrathin cell sections (c) showing electron-lucent PHB granules; GC-MS chromatogram (d) of biomass extracts (1, methyl 3-hydroxybutanoate; 2, methyl benzoate, internal standard).

Nile blue staining revealed numerous blue-stained granules in *P. endophytica* UCM B-5715^T^ cells (Figure 1a), which exhibited bright orange fluorescence under UV illumination (Figure 1b), indicating the presence of intracellular PHB inclusions (Ostle and Holt, 1982; Kharkhota et al., 2024). TEM revealed cytoplasmic regions densely filled with electron-lucent spherical to oval granules (Figure 1c), ranging in size from 65 × 48 nm to 1544 × 1127 nm, with average dimensions of 379 ± 267 × 286 ± 191 nm. Most granules ranged from 236 × 185 nm to 479 × 341 nm, with approximately 1–10 granules observed per cell. Gas chromatography–mass spectrometry (GC-MS) analysis confirmed that *P. endophytica* UCM B-5715^T^ produces poly-β-hydroxybutyrate (PHB), as indicated by a prominent peak at 3.24 min corresponding to the methyl ester of 3-hydroxybutanoic acid according to the National Institute of Standards and Technology (NIST02) database (Figure 1d).

### 3.2. Morphological features of isolated PHB granules

**Figure 2:**
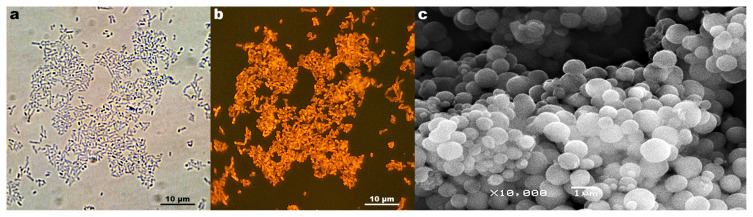
Light (a) and fluorescence (b) micrographs of Nile blue-stained PHB granules and SEM images (c) of PHB granules isolated from *P. endophytica* UCM B-5715^T^ cells.

Light microscopy of isolated PHB granules stained with Nile blue revealed blue-stained structures (Figure 2a) similar to those observed in intact cells (Figure 1a). Under UV illumination, the granules exhibited bright orange fluorescence (Figure 2b), consistent with the observations obtained from intact cells (Figure 1b). SEM revealed that the isolated granules exhibited a more rounded morphology (Figure 2c) than those observed in ultrathin cell sections (Figure 1c), with length-to-width ratios of 1.1–1.2 and 1.2–1.4, respectively, likely due to elongation artifacts associated with resin-embedded cells during TEM analysis. Granule dimensions exhibited substantial variability, with average dimensions of 626 ± 250 × 566 ± 239 nm, minimum dimensions of 72 × 42 nm, maximum dimensions of 1481 × 1350 nm, and the majority of granules ranging from 397 × 340 nm to 879 × 785 nm. The larger average granule size observed after isolation likely reflects the preferential loss of smaller granules during the purification procedure.

### 3.3. Physicochemical characterization of PHB

**Figure 3:**
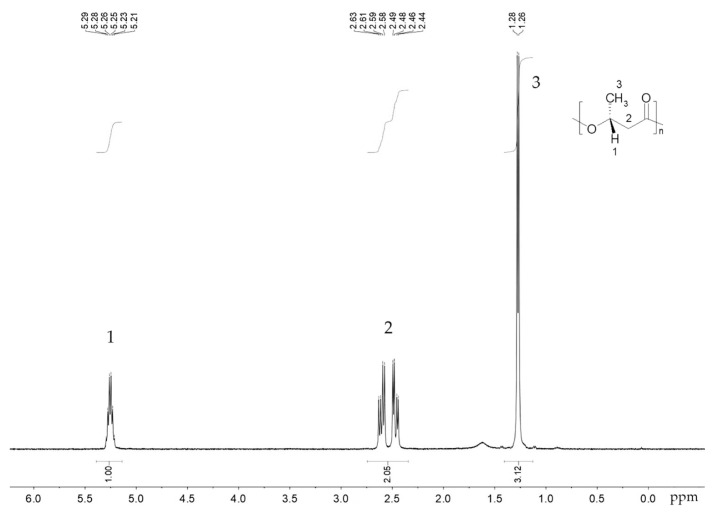
^1^H NMR spectrum of PHB produced by *P. endophytica* UCM B-5715^T^.

^1^H NMR spectroscopy of PHB (Figure 3) revealed characteristic signals corresponding to −CH at 5.25 ppm (sextet, J = 6.5 Hz), −CH_2_ at 2.47 ppm (dd, J = 15.5 and 7.4 Hz) and 2.6 ppm (dd, J = 15.5 and 5.8 Hz), and −CH_3_ at 1.27 ppm (d, J = 6.3 Hz), consistent with previously reported PHB spectra and confirming the polymer structure (Sindhu et al., 2011; Trakunjae et al., 2021).

**Figure 4:**
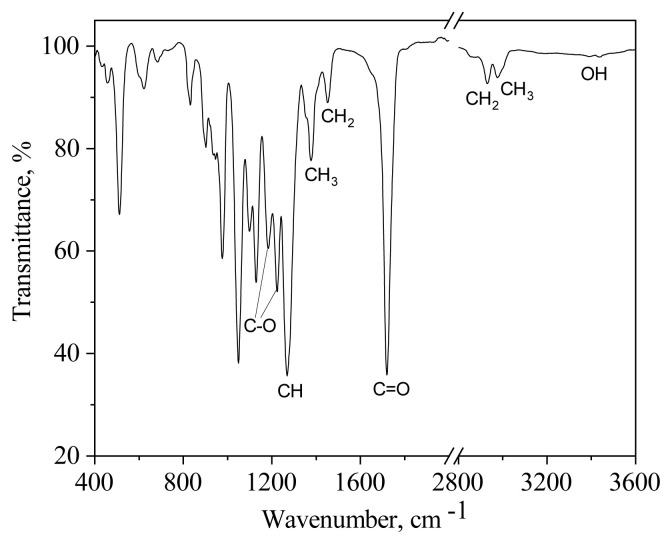
FTIR spectrum of PHB produced by *P. endophytica* UCM B-5715^T^.

The FTIR spectrum of PHB produced by *P. endophytica* UCM B-5715^T^ (Figure 4) exhibited a strong C=O stretching band at 1720 cm^−1^, characteristic C–O stretching bands at 1180 and 1227 cm^−^^1^, and a weak −OH stretching band at 3442 cm^−^^1^, consistent with previous reports (Trakunjae et al., 2021). Weak −CH_3_ and −CH_2_ stretching vibrations were observed at 2976 and 2932 cm^−^^1^, whereas bending vibrations associated with −CH_2_, −CH_3_, and −CH groups were detected at 1452, 1379, and 1275 cm^−1^.

**Figure 5:**
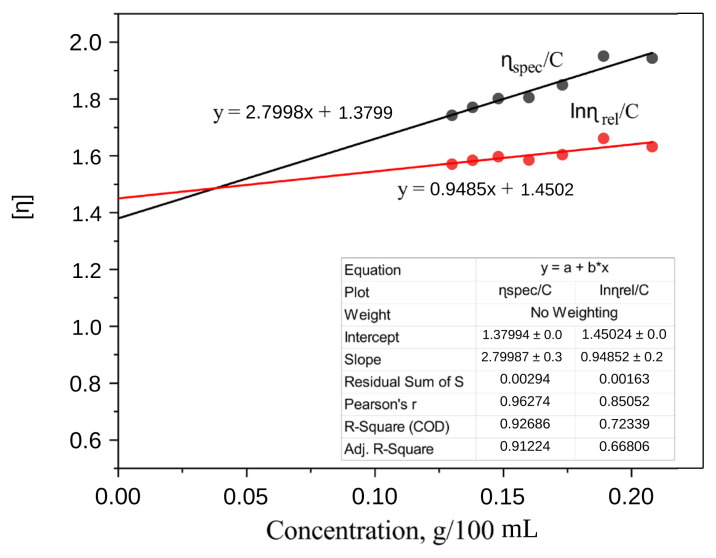
Reduced viscosity and inherent viscosity plots as a function of PHB concentration.

Figure 5 presents the plots of reduced viscosity (η_spec_/C) and inherent viscosity (ln(η_rel_)/C) as a function of PHB concentration. The intrinsic viscosity [η], determined from the intercepts of the reduced and inherent viscosity plots extrapolated to zero concentration, was 1.41 dL/g, corresponding to a viscosity-average molecular weight (M_v_) of approximately 170 kDa according to the Mark–Houwink equation.

**Figure 6:**
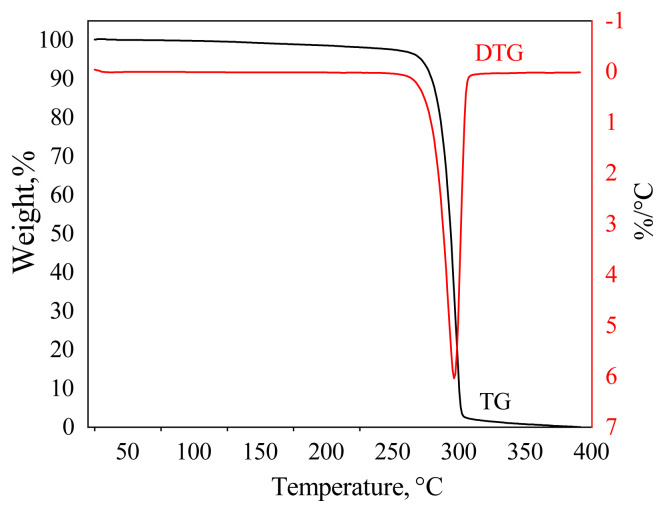
TGA and DTG curves of PHB produced by *P. endophytica* UCM B-5715^T^ under an air atmosphere.

TGA results (Figure 6; Table 1) demonstrated that PHB produced by *P. endophytica* UCM B-5715^T^ underwent single-step thermal degradation, with the onset of decomposition occurring at 271 °C (corresponding to 5% weight loss) and the maximum rate of mass loss observed at 296 °C.

**Figure 7:**
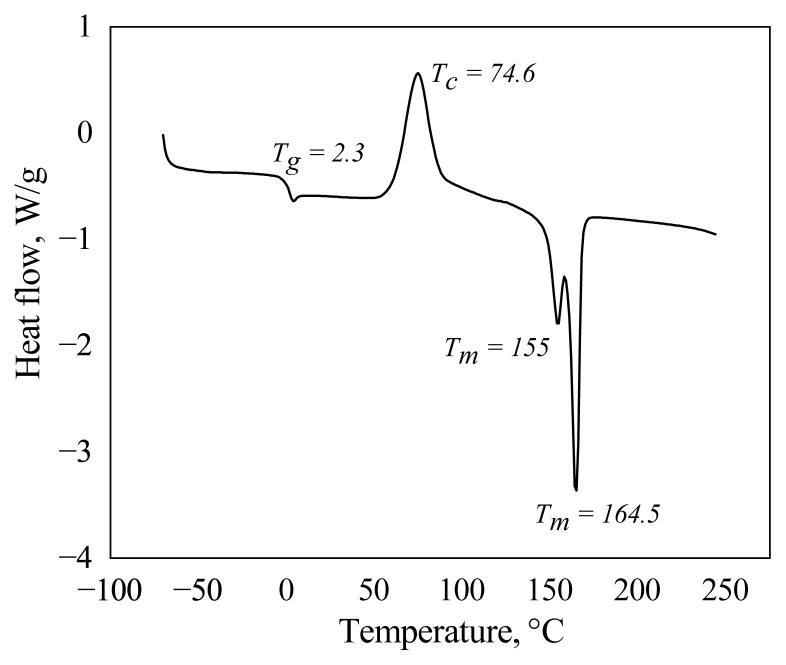
DSC thermogram of the PHB sample produced by *P. endophytica* UCM B-5715^T^ during the second heating cycle.

The DSC thermogram of PHB (Figure 7) exhibited two endothermic melting peaks at 155 and 164.5 °C, consistent with previously reported PHB thermal profiles (Sindhu et al., 2011; Wellen et al., 2013). The higher-temperature peak corresponds to the melting of more ordered crystalline domains, whereas the lower-temperature peak is associated with the melting of smaller and less perfect crystalline regions (Sindhu et al., 2011). The total melting enthalpy (ΔH_m_) was determined to be 77.3 J/g. A broad exothermic peak observed at 74.6 °C during the second heating cycle was attributed to melt crystallization, with an associated enthalpy change of ΔH = 59.5 J/g. The glass transition temperature (T_g_) was determined to be 2.3 °C, with a corresponding heat capacity change (ΔC_p_) of 0.56 J/(g·°C).

### 3.4. Genome characterization and genome mining of *P. endophytica*

The complete genome of *P. endophytica* UCM B-5715^T^ (= DSM 13796; GenBank accession no. GCA_900115845.1) comprises 5.1 Mbp and contains 38 scaffolds, 8 spanned gaps, and 5253 predicted genes, including 5144 coding DNA sequences, 17 rRNA genes, and 48 tRNA genes. Among the 12 publicly available *P. endophytica* strains, genome sizes ranged from 5.11 Mbp for DSM 13796^T^ to 5.8 Mbp for strain G25-135-1, whereas the numbers of protein-coding genes ranged from 4896 in strain KCTC 13922 to 5462 in strain FH5. All analyzed genomes exhibited similar G + C contents. A summary of the genomic features of the 12 *P. endophytica* strains is presented in Table 2.

**Figure 8:**
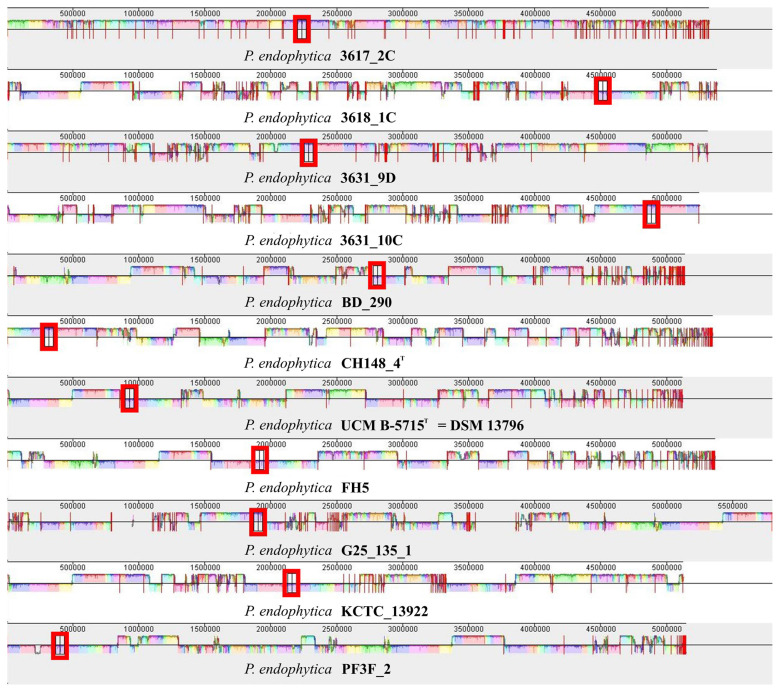
Whole-genome alignment of *P. endophytica* strains generated using progressiveMauve. (*pha* loci are highlighted in red)

Genomes assigned an “inconclusive” taxonomy check status were excluded from further analyses. Whole-genome alignment was performed using progressiveMauve (Figure 8). Among all protein-coding genes identified across the 11 genomes chosen for analysis, 3512 genes were found to possess at least one ortholog in the remaining genomes. The resulting alignment was subsequently screened to identify orthologous genes involved in PHA biosynthesis.

### 3.5. PHA biosynthetic gene clusters in *P. endophytica*

Genome mining of *P. endophytica* DSM 13796^T^ identified a *pha* locus containing the *phaP* (PHB inclusion protein), *phaQ* (transcriptional regulator), *phaR* (PHA synthase subunit), *phaB* (acetoacetyl-CoA reductase), and *phaC* (PHA synthase subunit) genes. Orthologs of these genes were identified in the other *P. endophytica* genomes through progressiveMauve alignment analysis (Table 3).

**Figure 9:**
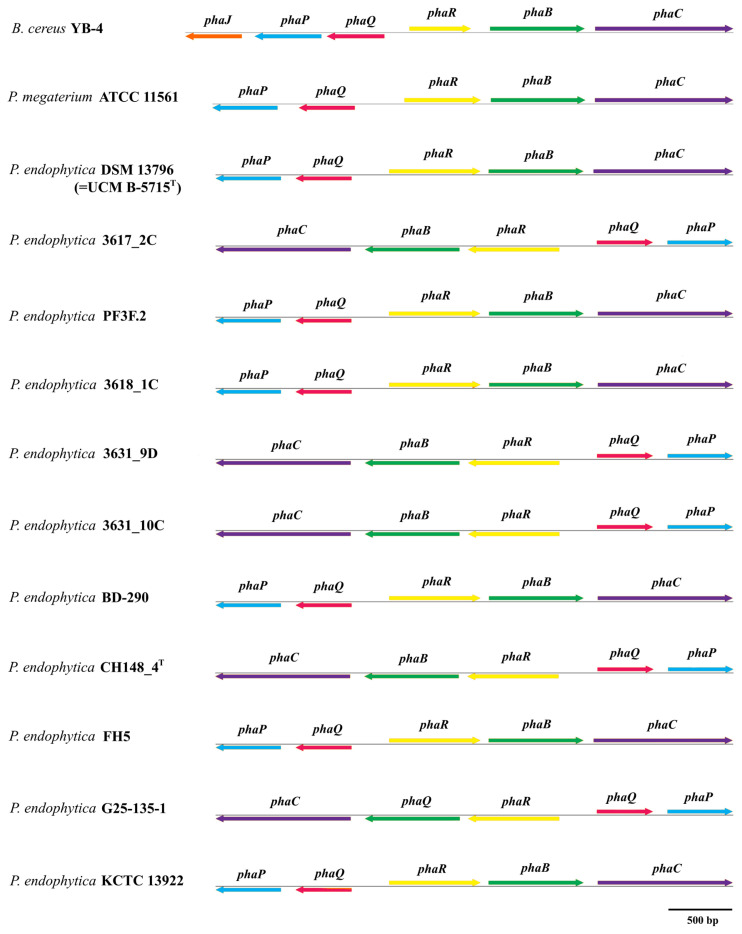
Multiple sequence alignment of the *pha* loci from *P. endophytica*, *P. megaterium* ATCC 11561, and *B. cereus* YB-4.

Multiple sequence alignment analysis performed using pyGenomeViz (Figure 9) demonstrated that these genes corresponded to those previously identified in the *phaR–phaB–phaC* and *phaP–phaQ* operons of *P. megaterium* ATCC 11561 (Lee et al., 2004). In contrast, the *pha* locus differed from that of *B. cereus* YB-4 (Kihara et al., 2017) because orthologs of the *phaJ* gene were absent.

**Figure 10:**
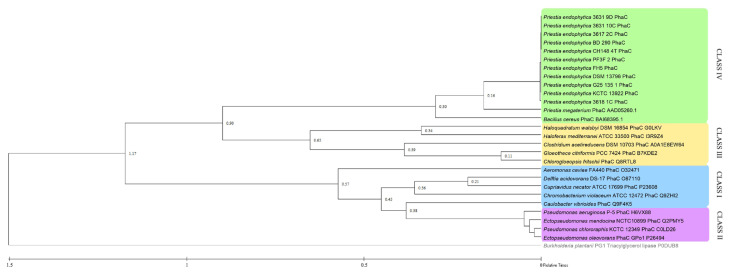
Maximum likelihood phylogenetic analysis and timetree reconstruction of PhaC proteins.

A phylogenetic tree based on 28 PhaC protein sequences from *P. endophytica* and other taxa was constructed using the maximum likelihood method and the JTT substitution model (Figure 10). The PhaC proteins of *P. endophytica* clustered with those of *P. megaterium* and *B. cereus*, forming a distinct Class IV PHA synthase clade. Relative divergence estimates among *P. endophytica* paralogs ranged from 0.11 to 0.30, indicating limited evolutionary divergence. This clade was identified as sister to the *P. megaterium* and *B. cereus* lineage, with a relative divergence estimate of 0.30. A deeper evolutionary split (relative divergence = 0.65) separated the Class IV clade from the Class III clade, which included PhaC sequences from *Chlorogloeopsis fritschii* (Q8RTL8), *Clostridium acetireducens* (A0A1E8EW64), *Gloeothece citriformis* (B7KDE2), *Haloferax mediterranei* (I3R9Z4), and *Haloquadratum walsbyi* (G0LKV6). The relative divergence estimate between the Class I/II clades and the Class III/IV clades was 1.17. Class I included *Aeromonas caviae* (O32471), *Caulobacter vibrioides* (Q9F4K5), *Chromobacterium violaceum* (Q9ZHI2), and *Delftia acidovorans* (O87110), whereas Class II included *Pseudomonas aeruginosa* (H6VX88), *Pseudomonas chlororaphis* (C0LD26), *Ectopseudomonas mendocina* (Q2PMY5), and *Ectopseudomonas oleovorans* (P26494).

### 3.6. PHB biosynthetic pathway in P. endophytica UCM B-5715^T^

**Figure 11:**
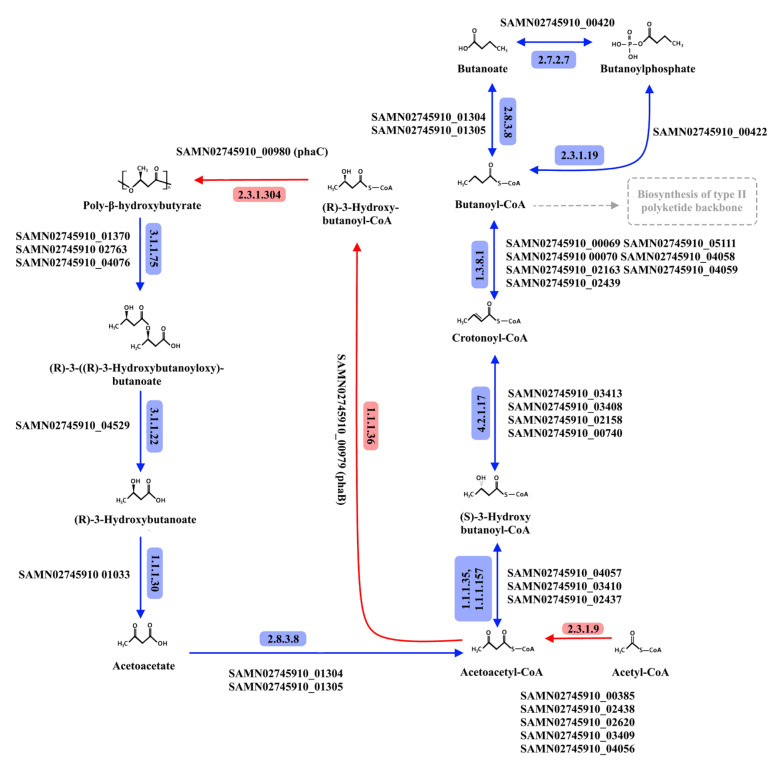
Reconstruction of the butanoate metabolic pathway in *P. endophytica* UCM B-5715^T^.

According to the metabolic reconstruction generated using the *merlin* platform, PHB biosynthesis and degradation in *P. endophytica* UCM B-5715^T^ (Figure 11) begin with the condensation of two acetyl-CoA molecules catalyzed by acetyl-CoA C-acetyltransferase (EC 2.3.1.9), resulting in the formation of acetoacetyl-CoA. Acetoacetyl-CoA is stereospecifically reduced by acetoacetyl-CoA reductase (EC 1.1.1.36) to form (R)-3-hydroxybutyryl-CoA, which is subsequently polymerized by PhaC (EC 2.3.1.304) through transesterification, resulting in PHB chain elongation and CoA release. PHB degradation is initiated by PHB depolymerase (EC 3.1.1.75), which generates oligomers and (R)-3-((R)-3-hydroxybutanoyloxy) butanoate. These intermediates are subsequently hydrolyzed by hydroxybutyrate dimer hydrolase (EC 3.1.1.22) to produce (R)-3-hydroxybutanoate, which is then oxidized to acetoacetate by 3-hydroxybutyrate dehydrogenase (EC 1.1.1.30). Acetoacetate is subsequently converted back to acetoacetyl-CoA by acetoacetyl-CoA:acetate CoA-transferase (EC 2.8.3.8). (S)-3-hydroxybutyryl-CoA may also enter the β-oxidation pathway through the activity of 3-hydroxyacyl-CoA dehydrogenases (EC 1.1.1.35 and 1.1.1.157). Downstream metabolites derived from butanoyl-CoA are processed through reactions involving enoyl-CoA hydratase (EC 4.2.1.17) and butyryl-CoA dehydrogenase (EC 1.3.8.1), ultimately leading to the formation of butanoic acid. Subsequently, butanoic acid is converted to butanoyl phosphate through the activities of butyrate kinase (EC 2.7.2.7) and phosphate butyryltransferase (EC 2.3.1.19), thereby linking PHB metabolism to broader biosynthetic and energy-generating pathways.

## 4. Discussion

This study provides the first comprehensive physicochemical and genomic characterization of poly-β-hydroxybutyrate (PHB) synthesized by the type strain *Priestia endophytica* UCM B-5715^T^. Its FTIR spectrum exhibited characteristic PHB absorption bands, including the ester carbonyl stretching band at 1720 cm^−1^, C–O–C stretching vibrations at 1180 and 1227 cm^−^^1^, and aliphatic −CH_2_ and −CH_3_ stretching bands at 2932 and 2976 cm^−^^1^. These spectral features correspond closely to previously reported PHB profiles from *P. endophytica* (Madhusoodanan et al., 2021) and other members of the family *Bacillaceae* (Baikar et al., 2017). A broad −OH stretching band at 3442 cm^−^^1^ suggested the presence of terminal hydroxyl groups or residual moisture (Priyanka et al., 2020). ^1^H NMR analysis was also consistent with previously reported microbial PHB spectra, displaying characteristic signals at 1.27 ppm (−CH_3_), 2.47 and 2.60 ppm (−CH_2_), and 5.25 ppm (−CH). These chemical shift values are consistent with previously reported PHB spectra obtained from *P. endophytica*, *P. megaterium*, and *Bacillus* spp. (Baikar et al., 2017; Madhusoodanan et al., 2021).

The observed viscosity-average molecular weight (M_v_) of approximately 170 kDa was comparable to those reported for PHB produced by *P. megaterium* (Baikar et al., 2017) and higher than the values reported for *P. aryabhattai* PHB10 and several other *Bacillus* strains, which typically range from 38 to 75 kDa (Masood et al., 2015; Pillai et al., 2017). Such a molecular weight profile may support favorable biodegradability while maintaining adequate mechanical performance. DSC analysis revealed a glass transition temperature (T_g_) near 2 °C and dual melting peaks at 155 and 164.5 °C, which may indicate the presence of multiple crystalline domains or variations in crystal perfection. Similar thermal behavior has been reported for certain strains, including *P. megaterium* (Joyline and Aruna, 2019), which also exhibited multiple melting transitions, although at comparatively lower temperatures (136.41 °C and 158.04 °C). The observed melting temperatures were slightly below the upper limit of the commonly reported PHB melting range of 168–175 °C but remained consistent with the melting temperatures reported for PHB produced by *P. aryabhattai* (170 °C) (Pillai et al., 2017) and *B. aerophilus* RSL-7 (163.99 °C) (Sabapathy et al., 2019). The onset of thermal degradation at 271 °C and the maximum degradation temperature at 297 °C were comparable to the thermal stability profiles reported for *B. aerophilus* RSL-7 and *P. megaterium* JHA (Joyline and Aruna, 2019; Sabapathy et al., 2019) and fell within the broad thermal degradation range reported for PHB (220–290 °C) (Kervran et al., 2022). Overall, the physicochemical and thermal properties of the PHB characterized in this study suggest that it possesses sufficient structural stability and mechanical performance for potential biomedical applications.

All *P. endophytica* genomes possessed the conserved *phaR–phaB–phaC* operon together with the oppositely oriented *phaP–phaQ* operon, mirroring the classical *P. megaterium* PHB biosynthetic gene cluster (Lee et al., 2004; Tsuge et al., 2015). MUSCLE alignment analysis revealed conserved catalytic motifs within PhaC proteins across all analyzed strains, suggesting the preservation of enzymatic function. To the best of our knowledge, this study represents the first comprehensive reconstruction of the PHB biosynthetic gene cluster in *P. endophytica*. Metabolic pathway reconstruction suggested that PHB biosynthesis is closely associated with acetyl-CoA availability, consistent with previously proposed metabolic models (Liang et al., 2024). The degradation of stored PHB to butanoyl-CoA may contribute to type II polyketide biosynthesis pathways, which were previously investigated in this bacterium in relation to the production of chlorxanthomycin and resistomycin (Kharkhota et al., 2024).

## 5. Conclusion

This study characterized the physicochemical properties and biosynthetic features of PHB produced by *P. endophytica* UCM B-5715^T^. Fluorescence microscopy, TEM, and SEM confirmed the presence of intracellular PHB granules exhibiting spherical to oval morphologies consistent with previously reported PHA inclusions. Genome analysis revealed a complete and conserved *pha* gene cluster (*phaP, phaQ, phaR, phaB*, and *phaC*), whose organization and sequence characteristics corresponded to those observed in other *Priestia* species and positioned the PhaC enzyme within the Class IV PHA synthase lineage. Metabolic pathway reconstruction further demonstrated that all core enzymatic steps required for PHB biosynthesis and degradation were present, consistent with the canonical pathways described for gram-positive bacteria.

Overall, this work provides the first integrated characterization of the physicochemical properties of PHB together with the *pha* gene cluster and its associated metabolic architecture in *P. endophytica*, thereby establishing the type strain as a well-defined reference for future comparative genomic and metabolic engineering studies.

## Figures and Tables

**Table 1:** TGA parameters of the PHB film produced by *P. endophytica* UCM B-5715^T^.

Sample	Temperature corresponding to mass loss Δm, °C			
	T_Δm 5%_	T_Δm = 10%_	T_Δm = 20%_	T_Δm = 50%_
PHB	271	279	285	293

**Table 2:** General genomic features of *P. endophytica* strains.

Strain	Genome size (bp)	GC content, %	Genes (GenBank)	Protein-coding genes (GeneBank)	Assembly
UCM B-5715T = DSM 13796	5,118,421	36.5	5240	5144	IMG-taxon 2599185264 annotated assembly
KCTC 13922	5,121,484	36.5	5087	4896	ASM159082v1
PF3F.2	5,145,076	36.5	5216	5003	ASM2371234v1
3631_9D	5,311,808	36.5	5303	5093	ASM326991v1
FH5	5,366,783	36.5	5546	5462	ASM383330v1
G25-135-1	5,801,706	36.5	5758	5411	ASM1584596v1
BD-290	5,136,811	36.5	5171	5013	ASM3622306v1
3631_10C	5,243,706	36.5	5235	5046	ASM326994v1
CH148_4T	5,346,832	36.5	5416	5146	ASM892380v1
3618_1C	5,379,838	36.5	5345	5136	ASM326997v1
3617_2C	5,318,960	36.5	5291	5119	ASM326995v1
N12A3*	5,308,076	36.5	5444	5210	ASM2679014v1

Note:

* Taxonomy check status: “Inconclusive”.

**Table 3:** Orthologous genes involved in PHA biosynthesis identified in *P. endophytica* strains.

*P. endophytica* strains	Assigned function				
	polyhydroxyalkanoate synthase, PhaC	polyhydroxyalkanoic acid synthase, PhaR subunit	acetoacetyl-CoA reductase, PhaB	polyhydroxyalkanoic acid inclusion protein, PhaP	transcriptional regulator, PhaQ
3617_2C	A4R27_11150	A4R27_11160	A4R27_11155	A4R27_11170	A4R27_11165
3618_1C	A4U60_22470	A4U60_22460	A4U60_22465	A4U60_22450	A4U60_22455
3631_9D	A3863_11590	A3863_11600	A3863_11595	A3863_11610	A3863_11605
3631_10C	A3864_24355	A3864_24365	A3864_24360	A3864_24375	A3864_24370
BD_290	P4602_RS14135	P4602_RS14125	P4602_RS14130	P4602_RS14115	P4602_RS14120
CH148_4T	F8155_01565	F8155_01575	F8155_01570	F8155_01585	F8155_01580
DSM_13796	SAMN02745910_00980	SAMN02745910_00978	SAMN02745910_00979	SAMN02745910_00976	SAMN02745910_00977
FH5	FH5_02914	FH5_02912	FH5_02913	FH5_02910	FH5_02911
G25_135_1	ABD68_09415	ABD68_09425	ABD68_09420	ABD68_09435	ABD68_09430
KCTC_13922	AZF06_06225	AZF06_06215	AZF06_06220	AZF06_06205	AZF06_06210
PF3F_2	M4D59_02180	M4D59_02170	M4D59_02175	M4D59_02160	M4D59_02165

## Data Availability

All data generated or analyzed during this study are included in this published article.
